# Molecular Cloning, Bioinformatics, and Expression Analysis of the *NPR1* Homolog in Sesame (*Sesamum indicum* L.)

**DOI:** 10.3390/plants14233557

**Published:** 2025-11-21

**Authors:** Mingfeng Yan, Xiaolin Zhao, Xingshen Li, Zhenrui He, Juling Hua, Lingen Wei, Yang Sun, Chuanxu Wan, Shuijin Huang

**Affiliations:** 1 Institute of Plant Protection, Jiangxi Academy of Agricultural Sciences, Nanchang 330200, China; mfyan2016@126.com (M.Y.); 15005163911@163.com (X.Z.); lixinshen0@163.com (X.L.); zhenruihe@163.com (Z.H.); huajl2000@126.com (J.H.); sun2007yang@126.com (Y.S.); 2 Jiangxi Provincial Key Laboratory of Agricultural Non-Point Source Pollution Control and Waste Comprehensive Utilization, Jiangxi Academy of Agricultural Sciences, Nanchang 330200, China; 3 Soil Fertilizer and Resource Environment Institute, Jiangxi Academy of Agricultural Sciences, Nanchang 330200, China; lgw0021@163.com

**Keywords:** sesame, *Ralstonia solanacearum*, benzothiadiazole, NPR1, disease resistance

## Abstract

Sesame bacterial wilt, caused by the pathogen *Ralstonia solanacearum*, is a major constraint for continuous cropping. Deciphering the defense mechanisms of sesame is therefore essential to the development of novel and effective control strategies. The *Non-expressor of Pathogenesis-Related* 1 (NPR1) plays a key role in regulating salicylic acid (SA)-mediated systemic acquired resistance (SAR). In this study, we reported that leaf treatments with 50 μg/mL benzothiadiazole (BTH) resulted in increased protection of sesame against *Ralstonia solanacearum*. We clarified the structure, expression patterns, and function of a *NPR1* homologous gene, *SiNPR1*, in sesame. The *SiNPR1* gene open reading frame comprises 1758 bp, and it encodes 585 amino acids. Phylogenetic analysis revealed that SiNPR1 is closely related to NPR1-like in *Olea europaea* and clustered with other members of the families Monocotyledon and Dicotyledon. Quantitative real-time PCR (qRT-PCR) results demonstrated that the expression of the *SiNPR1* gene was organ-specific and could be induced by BTH. The yeast two-hybrid assay confirmed that SiNPR1 directly interacts with SiTGA2. In conclusion, these results suggest that SiNPR1 plays a pivotal role in the BTH-dependent systemic acquired resistance in sesame.

## 1. Introduction

Plants have evolved complex and precise immune systems to defend against pathogen attacks. The defense system is tightly regulated through induced responses by several plant hormones, particularly salicylic acid (SA) [1,2,3]. SA acts as a defense signaling molecule and is involved in the induction of the systemic acquired resistance (SAR) pathway [1,2]. SAR is a long-lasting and broad-spectrum resistance that protects the entire plant, and is typically activated upon local pathogen invasion [3,4,5]. When plants are attacked by pathogens, the concentration of SA increases from basal levels both locally and systemically to high levels [5,6]. Concurrently, SA activates some pathogenesis-related (*PR*) genes locally at the infection site and systemically in distant plant tissues [3,7]. Additionally, in many plants, applying exogenous SA or its functional analogs, such as 2,6-dichloroisonicotinic acid (INA), 3,5-dichloroanthranilic acid (DCA), N-cyanomethyl-2-chloroisonicotinic acid (NCI), and benzothiadiazole (BTH), can also induce the SAR and enhance disease resistance [8,9,10].

The *Non-expressor of Pathogenesis-Related 1* (*NPR1*) gene acts as a transcription coactivator in the defense response and is involved in the SA-mediated SAR pathway, playing a crucial role in regulating plant overall disease resistance signaling [11,12]. Additionally, NPR1 participates in the plant’s response to various abiotic stresses, including salt, drought, and low temperatures [13]. NPR1 was first discovered in the model plant *Arabidopsis thaliana* mutants, and its mechanism of action has been well elucidated over nearly 30 years of research [14,15]. Studies have shown that NPR1 is an SA receptor protein located downstream of the SA signaling pathway. Its structure includes an N-terminal activation domain with BTB/POZ motifs, multiple ankyrin repeat domains in the middle, and a C-terminal transactivation domain known as NPR1-like_C [16,17]. In non-induced states, NPR1 exists in the cytoplasm as a multimer. Upon pathogen invasion, which increases SA levels, intracellular reduction potential causes the disulfide bond at position 156 of the multimeric NPR1 to break, which is transported into the nucleus to interact with multiple members of the TGA family, ultimately leading to the transcription of the *PR* gene [12,14,18]. This positively regulates the expression of early defense genes induced by SA, ultimately leading to the formation of systemic acquired resistance, granting plants broad-spectrum and long-lasting disease resistance [2]. With the advancement of whole-genome sequencing in plants, the *NPR1* gene family has been cloned and identified in various species, including rice, wheat, apple, citrus, soybean, cotton, tobacco, and kiwifruit [19,20,21,22,23]. Overexpression of the rice *OsNPR1* gene in rice increases resistance to bacterial leaf blight [24]. Similarly, in dicotyledonous plants, overexpression of the *NPR1* gene in citrus improves resistance to Huanglongbing and canker diseases [25]. Overexpression of an endogenous *NPR1* orthologue in apple increased resistance to fire blight and two other major fungal pathogens (*Venturia inaequalis* and *Gymnosporangium juniperi-virginianae*) of apple [26]. Furthermore, in wheat, the NPR1 protein regulates phenylpropanoid metabolism, affecting the synthesis of antimicrobial secondary metabolites such as flavonoids, isoflavonoids, and lignin [27]. The above studies demonstrate that similar defense mechanisms exist in many crop species, and manipulating the expression of *NPR1* or its homologous genes could effectively improve crop disease resistance [28].

Sesame (*Sesamum indicum* L.) has been an important oil crop in Asian countries since ancient times, renowned for its abundant oil and protein content in its seeds [29]. The sesame seed has been considered the “queen of oilseeds” because of its high oil content (35–60%) and quality [29,30]. Currently, the global cultivation area spans approximately 12.84 million hectares, with an annual production of around 6.74 million tons [29,31]. However, sesame bacterial wilt, caused by the bacterial pathogen *Ralstonia solanacearum*, severely threatens sesame production [32]. Unfortunately, effective prevention and control methods for this disease are lacking. China is both a major producer and consumer of sesame. Most sesame varieties grown in China are susceptible or moderately susceptible to bacterial wilt, and no highly resistant germplasm resources have been discovered yet [32,33,34]. Additionally, our knowledge concerning sesame defense is still limited.

In our previous study, four sesame cultivars (Jinhuangma, Poyang Heizhima No. 5, Ganzhi No. 5, and Yuzhi No. 11) with moderate resistance to *R*. *solanacearum* were identified from 29 major Chinese varieties [33]. This study revealed that foliar application of benzothiadiazole (BTH), a SA analog, significantly reduced bacterial wilt incidence across four sesame cultivars. Notably, Poyang Heizhima No. 5 exhibited the most substantial enhancement in resistance compared to other cultivars. To investigate the role of NPR1 during BTH-induced resistance, we isolated and characterized *SiNPR1* as a key regulator mediating BTH-induced defense responses in *R*. *solanacearum*-resistant sesame. Based on the nucleotide and the amino acid sequences of *SiNPR1*, we analyzed its bioinformatics, subcellular localization, and the organ-specific expression patterns of this gene and its dynamic responses to BTH treatments by RT-qPCR. Additionally, the importance of the BTB/POZ domain of SiNPR1 was evaluated using the yeast two-hybrid system. This work provides the basis for further studies of SAR in sesame.

## 2. Results

### 2.1. BTH Elicitation on the Inhibition of Ralstonia Solanacearum Infection in Sesame

**Figure 1:**
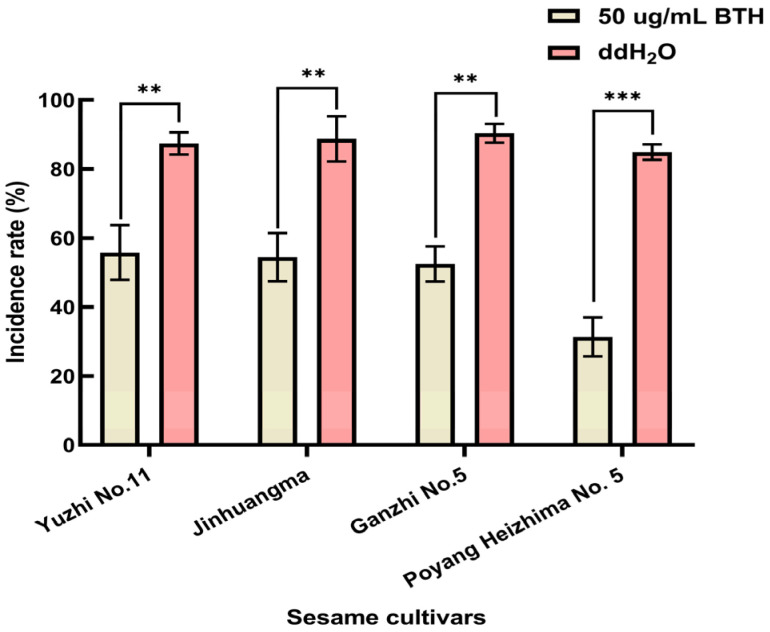
Changes in disease incidence in four sesame cultivars with or without 50 µg/mL BTH treatment (**: 0.001 < *p* < 0.01, ***: 0.0001 < *p* < 0.001).

BTH is one of the most commonly used plant inducers, which has been proven to be effective in inducing resistance to bacterial, fungal, and viral infections in a variety of plants, such as tobacco, cucumber, apple, wheat, etc [35,36,37,38]. In this study, the efficacy of BTH in enhancing sesame resistance against *Ralstonia solanacearum* infection was evaluated. Compared to the H_2_O-treated control group, foliar application of 50 μg/mL BTH significantly reduced disease severity in sesame plants three weeks post-inoculation. Notably, the cultivar “Poyang Heizhima No. 5” exhibited the most pronounced improvement in resistance, with a remarkable 63.09% reduction in disease incidence (Figure 1). These results demonstrate that BTH pre-treatment effectively primes sesame plants to combat *R*. *solanacearum* infection. Furthermore, the observed variation in disease suppression among different sesame cultivars highlights genotype-dependent differences in the induction of systemic resistance by BTH.

### 2.2. Gene Cloning and Sequence Analysis of SiNPR1

**Figure 2:**
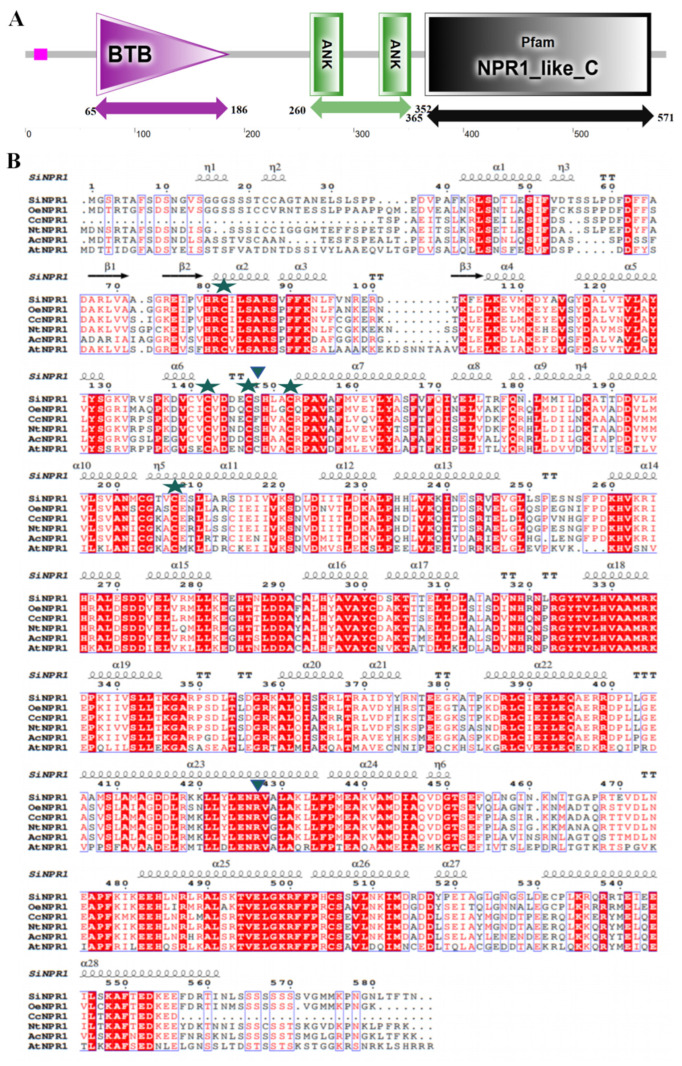
The structure of sesame SiNPR1. (**A**) A schematic representation of the SiNPR1 after analyzed by SMART. (**B**) Multiple alignment of SiNPR1 with NPR1-like proteins. Asterisks (five-pointed star) label the five conserved cysteine residues among the proteins. An inverted triangle marks the Cys^156^ and Arg^432^ relative to *Arabidopsis*. The mature protein sequences were used for the analysis. Their accession numbers are OeNPR1 (CAA3012024), CcNPR1 (CAP12787), NtNPR1 (AAM62410), AcNPR1 (PSS20797), and AtNPR1 (NP_176610).

It has been previously reported that BTH up-regulates the expression of *NPR1* or its homologous genes, thereby activating defense responses in plants [24,38]. In the next step, the RACE-PCR method was used to obtain full-length transcripts of the *NPR1* homolog *SiNPR1* (GenBank accession no. PX427686) from the sesame variety “Poyang Heizhima No. 5” (Figure S1). A NCBI-BLAST homology search revealed that the deduced amino acid sequence of *SiNPR1* shared high similarity to a broad range of different plant species, such as *Paulownia fortunei* PfNPR1 (89.1%) and *Solanum lycopersicum* LeNPR1 (73.2%). The basic physicochemical properties of the SiNPR1 protein were analyzed through ExPASy. The full-length *SiNPR1*, containing a 1758 bp ORF (open reading frame) region, encodes a protein of 585 amino acids with an estimated molecular weight of 65.03 kDa, an average hydrophilicity of −0.167, a lipid coefficient of 94.02, and an isoelectric point (pI) of 5.77. The prediction of the conserved structural domains of the SiNPR1 protein was analyzed through SMART. The SiNPR1 protein also shared conserved domains with other known NPR1 homologs: a BTB/POZ domain (amino acids 65 to 186), two ankyrin repeat domains (amino acids 260 to 352), and an NPR1/NIM1-like defense protein C-terminal motif (amino acids 363 to 571) (Figure 2A). A BLAST homology search revealed that the SiNPR1 protein exhibits high similarity to various eudicot plant species, such as *Paulownia fortunei*, *Nicotiana tabacum*, *Rehmannia glutinosa*, *Capsicum chinense*, *Gossypium hirsutum*, *Olea europaea*, and *Actinidia chinensis*.

Previous studies demonstrated that the five cysteine residues (Cys^82^, Cys^150^, Cys^155^, Cys^160^, and Cys^216^) located within the active center cavity are highly conserved among all the sequences, and these residues are proposed to be involved in the oligomer-monomer transition of NPR1 or its ortholog [12,39]. Specifically, Cys^156^ promotes oligomerization of NPR1 in vivo through S-nitrosylation, while Arg^432^ in the C-terminus is responsible for SA binding in *Arabidopsis thaliana* NPR1 (AtNPR1) [12]. A multiple alignment using ClustalW showed that these five highly conserved cysteine residues are required for the oligomer monomer conversion of AtNPR1 and are also present in SiNPR1 (Figure 2B). The SiNPR1 protein harbored conserved arginine residues at R^432^. However, functionally relevant residue Cys^156^, which is associated with oligomerization, is absent in SiNPR1 (Figure 2B). These substitutions may result in differences in substrate recognition between this sequence and others.

### 2.3. Structural Analysis of the SiNPR1 Protein

**Figure 3:**
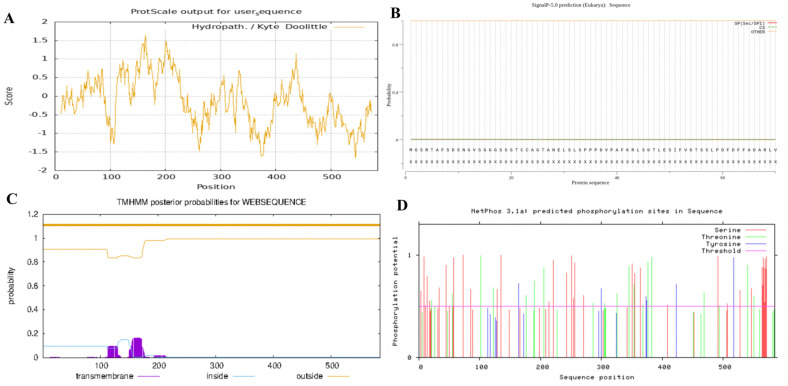
Prediction of hydrophobicity, transmembrane helix, signal peptide, and phosphorylation of the SiNPR1 protein. (**A**) Prediction of hydrophobicity or hydrophilicity. (**B**) Signal peptides and their site prediction on proteins. (**C**) Prediction of transmembrane structural domains. (**D**) Phosphorylation site prediction.

The results of the ProtScale online software analysis showed that the protein had a distinct hydrophilic region and predicted that the SiNPR1 protein was hydrophilic (Figure 3A). SignalP 4.1 analysis revealed that the SiNPR1 protein did not contain a signal peptide and was a non-secreted protein (Figure 3B). The TMHMM server V2.0 prediction revealed that SiNPR1 is located outside the membrane (Figure 3C). The phosphorylation checkpoint of the SiNPR1 protein was analyzed by NetPhos 3.1, and it was found that the SiNPR1 protein has potential phosphorylation checkpoints of tyrosine (Tyr), serine (Ser), and threonine (Thr) (Figure 3D).

**Figure 4:**
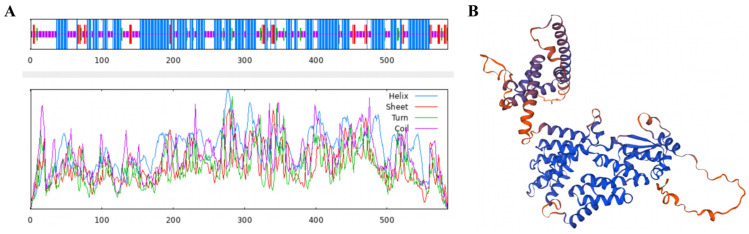
The secondary and tertiary structure prediction of the SiNPR1 protein. (**A**) Secondary structure prediction of SiNPR1. Alpha helix (blue); Random coil (purple); Extended strand (red); Beta turn (green). (**B**) Predicted tertiary structure prediction of SiNPR1. Structural models generated by homology modeling using SWISS-MODEL.

The secondary structure prediction of the SiNPR1 protein was performed using SOPMA online software. The results showed that the secondary structure of SiNPR1 mainly consisted of four forms, including 53.16% alpha-helix, 34.02% irregular curl, 8.03% extended chain, and 4.79% beta-fold (Figure 4A). The subcellular prediction showed that the SiNPR1 protein was most likely located in the nucleus, with a score of 0.789 (Table S1). Using SWISS-MODEL to predict the tertiary structure of the SiNPR1 protein, it was found that the predicted results of the tertiary structure of the SiNPR1 protein were consistent with the predicted results of the secondary structure of the protein. The sequence agreement with the template protein (PDB number: A0A5J5AV64.1.A) was 75.96%, the coverage was 100%, and the GMQE value was 0.75 (Figure 4B). The SiNPR1 protein belongs to the NPR1-like family, and the spatial structure is dominated by α-helix and random coiling; these findings are consistent with the predicted results of the secondary structure.

### 2.4. Phylogenetic Analysis of the SiNPR1 Protein

**Figure 5:**
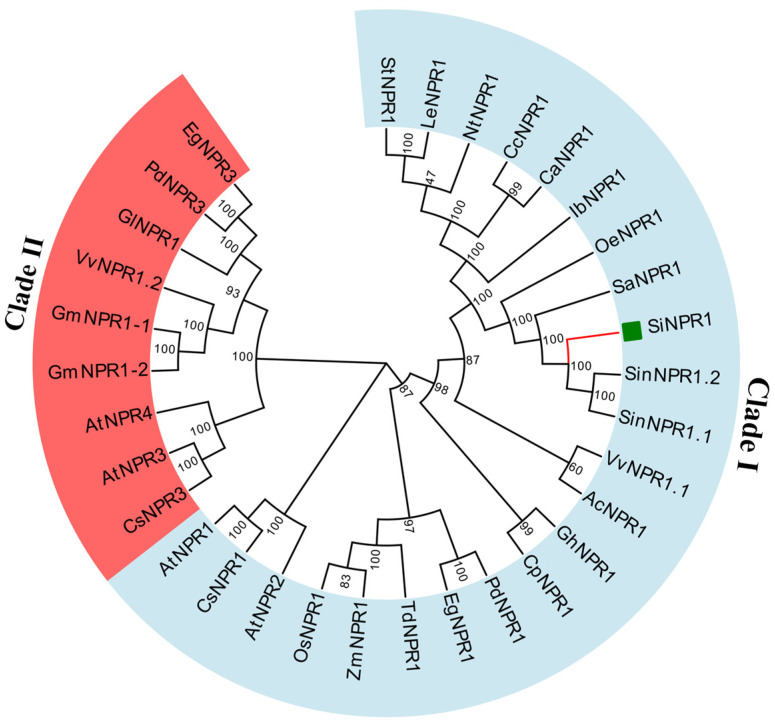
Phylogenetic relationship of the NPR1-like proteins from sesame and other different plant species. The tree was generated with the MEGA11 software (Version 11.0.13) using the neighbor-joining method with 1000 bootstrap replicates. The branch where SiNPR1 is located is represented by a red line, and SiNPR1 is represented by a square. The accession numbers and species names of all the NPR1-like proteins utilized in the analysis were summarized in Supplementary Table S2.

To further understand the evolutionary relationships between SiNPR1 and other plant NPR1 proteins, the NJ method using MEGA11.0 software was used to construct a phylogenetic tree, and all of the species were divided into two major clades, including clade I, and II. As shown in Figure 5, SiNPR1 was the most closely related to OeNPR1, CaNPR1, CcNPR1, and NtNPR1. SiNPR1 was classified into clade I, including AtNPR1, AcNPR1, and NtNPR1, which were reported as positive regulators of SAR. Clade II, including AtNPR3 and AtNPR4, served as negative regulators of SAR. The data suggest that SiNPR1 is likely a homolog of NPR1 in sesame and may function as a positive regulator of SAR.

### 2.5. Subcellular Localization of the SiNPR1 Protein

**Figure 6:**
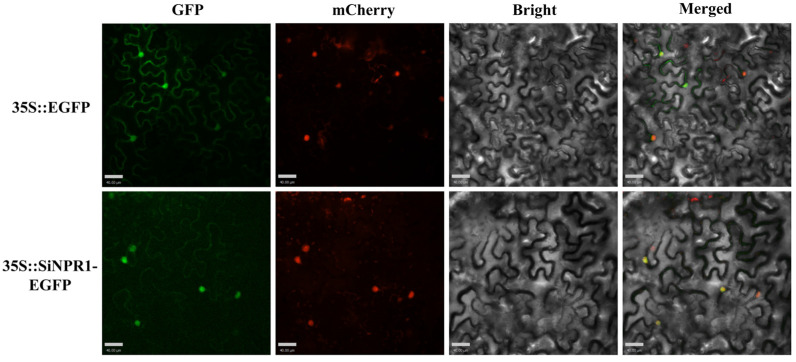
Subcellular localization of SiNPR1 protein in *N*. *benthamiana*. The constructs of 35S::SiNPR1-EGFP and blank control 35S::EGFP were transiently expressed in *N*. *benthamiana* leaf epidermal cells, respectively. Fluorescence signals were examined using a laser confocal scanning microscope. Scale bars = 40 μm.

To determine the subcellular localization of SiNPR1, the coding region of SiNPR1 (stop codon removed) was ligated to the 5′ end of the enhanced green fluorescent protein (EGFP) reporter gene, and the fragments were inserted into the plasmid vector pCAMBIA1302. Subsequently, the fusion construct SiNPR1-EGFP was introduced into 3–4-week-old tobacco leaves. The samples were then treated in the dark for 72 h, and GFP fluorescence was observed under a confocal fluorescent microscope. The results indicate that the fluorescence of SiNPR1-EGFP is primarily observed in the nucleus (Figure 6). Therefore, this is consistent with the bioinformatics analysis results, which show that SiNPR1 primarily functions as a nuclear protein in these cell types.

### 2.6. Cis-Elements Analysis of the SiNPR1 Promoter

**Figure 7:**
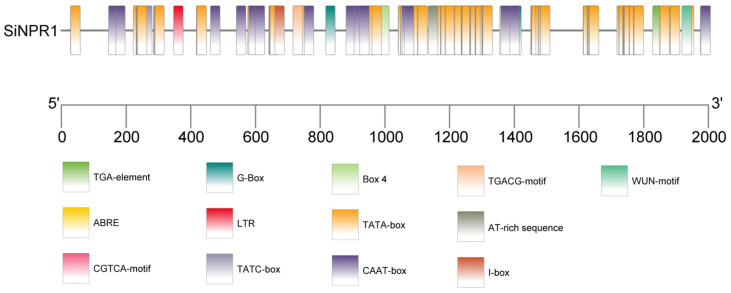
Schematic illustration of predicted *cis*-regulatory elements in the *SiNPR1* promoter region. The colored shapes represent different *cis*-regulatory elements as indicated.

The 2.0 kb upstream promoter region of the *SiNPR1* gene from the sesame variety Poyang Heizhima No. 5 was analyzed with PlantCARE online tool to search the *cis*-elements. As shown in Table S3, 133 *cis*-regulatory elements of 13 different types were identified within the SiNPR1 promoter sequence, including one TATC-box, 16 CAAT-box, and 102 TATA-box basal elements. In addition, we also identified the presence of several cis-acting elements related to phytohormone and abiotic stress factors regulation, such as the ABRE, TGA-element, CGTCA-motif, GARE-motif, G-Box, and LTR (Figure 7), which are recognized to participate in abscisic acid, auxin, methyl jasmonate (MeJA), gibberellin, light, and low-temperature-associated responses, respectively.

### 2.7. Expression Profile of SiNPR1 in Various Organs and in Response to BTH Treatments

**Figure 8:**
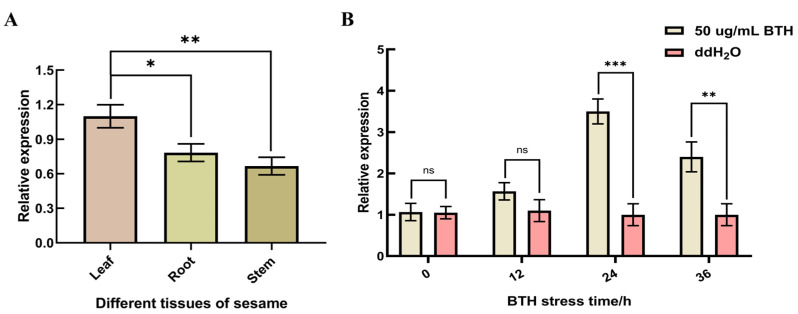
Expression patterns of *SiNPR1* in sesame. (**A**) Expression patterns of *SiNPR1* in different organs using qRT-PCR. Expression levels of *SiNPR1* in leaves, roots and stems. (**B**) Expression level of *SiNPR1* in leaves in response to BTH. (ns: no significance, *: *p* < 0.05, **: 0.001 < *p* < 0.01, ***: 0.0001 < *p* < 0.001).

To further explore the function of SiNPR1, this study examined its expression patterns in various organs and under different treatment conditions using quantitative real-time RT-PCR analyses. The expression of *SiNPR1* in various organs is indicated in Figure 8A, the transcripts of *SiNPR1* were detected in nearly all of the organs investigated, while the results revealed significant differences in expression levels between leaves, stems, and roots. The *SiNPR1* expression levels in stems and roots were significantly lower than those in leaves, suggesting that *SiNPR1* was constitutively expressed.

Previous reports have indicated that exogenous plant defense molecules, such as SA or BTH, can induce expression of *NPR1* or its homologous genes, thereby activating plant disease resistance responses [36,37]. In this study, we analyzed the temporal expression levels of *SiNPR1* in leaves following treatment with BTH using quantitative real-time RT-PCR. As shown in Figure 8B, when applying 50 μg/mL BTH treatment, the gene expression level of *SiNPR1* was significantly induced, reaching its highest level 24 h after treatment.

### 2.8. SiNPR1 Could Interact with SiTGA2

NPR1 has been suggested to interact with members of the TGA family of transcription factors, including TGA2 [18,19,40]. TGA2 is an SA-responsive and NPR1-dependent transcription activator [18,41]. TGA2 and NPR1 are activators of systemic acquired resistance (SAR) and of the SAR marker gene *pathogenesis-related-1* (*PR-1*) in *Arabidopsis thaliana* [18,41,42].

**Figure 9:**
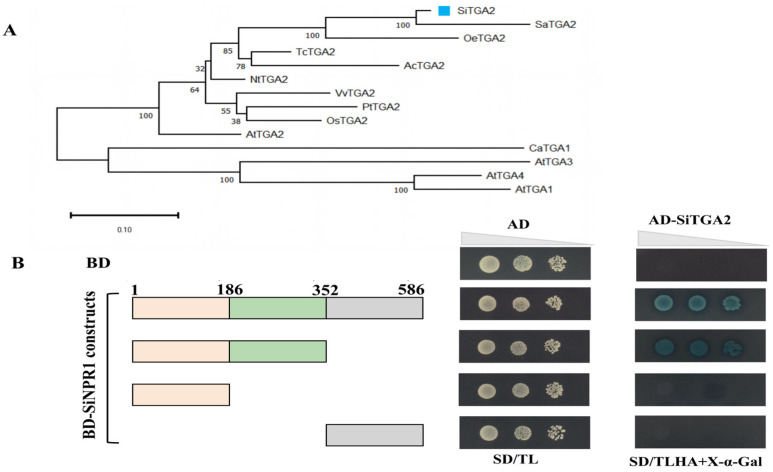
SiNPR1 interacted with SiTGA2. (**A**) Phylogenetic relationship of SiTGA2 with other TGA proteins from other plants. The tree was generated with the MEGA11 software (Version 11.0.13) using the neighbor-joining method with 1000 bootstrap replicates. SiNPR1 is indicated by a square. The accession numbers and species names of all the TGA homolog proteins used in the analysis were summarized in Supplementary Table S4. (**B**) Interaction assay between SiNPR1 and SiTGA2 using the yeast two-hybrid system. Transformed yeast cells were diluted with 0.9% NaCl into three concentration gradients (10^−1^, 10^−2^, and 10^−3^), and streaked on SD/TLHA (SD/-Trp/-Leu/-His/-Ade) medium containing 20 mg/mL X-α-Gal.

Here, the *SiTGA2* full-length cDNA sequence was obtained and used to determine whether it could interact with the SiNPR1 protein. The resulting phylogenetic tree indicated that SiTGA2 was clustered with other TGA2 homologs in plants (Figure 9A). TGA2 interacts with NPR1 to form an enhanceosome with transcriptional activation properties requiring the BTB/POZ domain of NPR1 [16,17,18]. To further explore the mode of action of SiNPR1, we investigated its interaction with TGA2 by yeast two-hybrid assay. The negative control (pGBKT7-SiNPR1 + pGAD-T7) and the empty vector experimental group (pGBKT7 + pGAD-T7) only grew on the SD/TL solid medium, indicating that SiNPR1 does not possess self-activating activity (Figure S2). As demonstrated in Figure 9B, both the full-length SiNPR1(1-582) and the SiNPR1(1-360) truncation, which included the BTB/POZ and ankyrin repeat domains, interacted with SiTGA2. The results indicated that SiNPR1 can interact with SiTGA2.

## 3. Discussion

Sesame is one of the oldest oil crops in the world [29]. However, the infestation and damage caused by *Ralstonia solanacearum* have become a significant limiting factor for achieving high and stable yields of sesame [32,34]. Understanding the mechanisms that regulate defense responses in sesame has profound implications for sesame breeding programs, which will help guide the screening of new sources of disease resistance in germplasm resources. Previous studies have confirmed that SAR is one of the primary defense pathways for plants to generate persistent resistance, which enables them to resist the invasion of pathogens [10,12]. SA or its analogue BTH can also stimulate the production of SAR [9,10]. As a core element in the regulation of the plant disease resistance signaling pathway, *NPR1* participates in the systemic disease resistance response of various plants by regulating the expression of disease course-related genes [12,14]. Although the *NPR1 homologue* has been isolated and characterized in several plants, the function of the *NPR1 homologue* in sesame remains poorly understood.

The NPR1 protein and its paralogues contain a bipartite nuclear localization sequence and two identifiable protein–protein interaction domains: BTB/POZ domains and ankyrin repeat domains [17]. In the present study, a novel full-length *NPR1*-like gene, designated *SiNPR1*, from sesame was characterized, and bioinformatics analysis was performed. Genetic structure analysis revealed that the SiNPR1 amino acid sequence had 69.1% homology with AtNPR1, and they had typical features, such as BTB/POZ domains, ankyrin repeat domains, and anchor protein repeats, which were highly conserved in the NPR1 protein of monocotyledonous and dicotyledonous plants (Figure 2A). Conservation of these structural domains in SiNPR1 indicated they might play a similar function and role in *Arabidopsis*.

In previous studies, it has been shown that Cys^82^, Cys^150^, Cys^155^, Cys^160^, and Cys^216^ are involved in the oligomer-monomer transition of NPR1 or NPR1-like proteins [12,39]. These five cysteine residues that are present in the BTB/POZ domain of NPR1 are conserved in SiNPR1 and AtNPR1 (Figure 2B). Previous studies demonstrated that in vivo via S-nitrosylation of Cys^156^ facilitates NPR1 oligomerization in *Arabidopsis* [12]. Interestingly, the Cys^156^ in SiNPR1 has been replaced by a serine residue (Figure 2B). However, studies on kiwifruit AcNPR1a have reported that although Cys^156^ is substituted in AcNPR1a, AcNPR1a still restores NPR1 function in the *Arabidopsis npr1-1* mutant, suggesting a potential role for other residues in preserving AcNPR1a protein homeostasis [19]. It is therefore plausible that other conserved residues within SiNPR1 also assume critical functions in the defense regulation of sesame. Furthermore, a potential nuclear localization signal was also identified within the C-terminal region of the SiNPR1 protein (Figure 2A). Phylogenetic analysis revealed that the SiNPR1 protein is most closely related to proteins from *Sesamum alatum*, *Olea europaea*, *Solanum tuberosum*, and *Nicotiana tabacum*, and is most distantly related to proteins from *Arabidopsis*, *Rice*, *Zea mays*, and *Triticum durum* (Figure 5). Meanwhile, SiNPR1 and AtNPR1 belong to the same lineage (clade I), which is essential for SAR establishment. The data indicate that the SiNPR1 protein shared some common characteristics with NPR1 homologs in other plants.

Nuclear localization of NPR1 is crucial for its function. Previous studies revealed that when the plant is not under biotic stress factors, NPR1 is mainly present in the cytosol as oligomers [43]. Upon SA induction, monomers are released from the oligomers and moved into the nucleus [12,43]. However, in this study, unlike AtNPR1, the transiently expressed SiNPR1-GFP fusion protein was predominantly located in the nucleus of *N*. *benthamiana* leaf cells (Figure 6). The observed nuclear translocation of SiNPR1 may be attributed to variations in species, infiltration procedures, or critical amino acid residues. Consistently, transiently expressed AeNPR1a-GFP and VvNPR1-GFP fusion proteins were localized predominantly to the nucleus, even in the absence of the SAR inducer SA induction [19,44]. This result suggests that SiNPR1 is a nuclear localization protein.

To investigate the regulatory mechanism of the *SiNPR1* gene expression, this study cloned a 2.0 Kb promoter of *SiNPR1* and identified several *cis*-acting regulatory elements. These elements include those responsive to plant hormones and defense mechanisms, indicating that the expression of the SiNPR1 gene is influenced by both plant hormones and stress factors (Figure 7). *SiNPR1* was constitutively expressed across various organs, with particularly high levels in the leaves (Figure 8), consistent with the expression patterns of *NPR1* homologs in *Sugarcane* and *Gladiolus hybridus* [45,46]. We also found that the expression of SiNPR1 could be induced by BTH. Previous studies have demonstrated that BTH activates plant defense responses and confers resistance to pathogens in tobacco, tomatoes, and bananas [47,48,49]. Similarly, our study revealed that BTH can enhance sesame’s resistance to *R*. *solanacearum*, although varietal differences were observed. We speculate that these variations may be associated with differences in promoter activity of NPR1 among cultivars, a hypothesis that warrants further investigation. Additionally, in *Arabidopsis*, NPR1 binds to the TGACG-binding factor (TGA) transcription factors, inducing the expression of downstream *PR* genes [18,41]. This interaction is crucial for activating defense genes and conferring resistance to secondary infections. Therefore, we investigated the potential interaction between SiNPR1 and SiTGA2 using a yeast two-hybrid assay. Our findings validate the interaction between these two proteins and further suggest the functional importance of the BTB/POZ and anchor protein repeat domains in the SiNPR1 protein (Figure 9). Whether the SiNPR1-SiTGA2 interaction regulates the transcription of PR genes in sesame and thereby triggers SAR remains to be validated.

In summary, the *NPR1* homolog, denoted *SiNPR1*, was characterized in sesame. The SA analogue BTH could induce sesame to defend against *R*. *solanacearum*, while the transcription level of *SiNPR1* was upregulated under BTH treatment. On the other hand, SiNPR1 was able to interact with SiTGA2. These results suggest that SiNPR1 plays an important role in the defense response of sesame, and further studies are needed to obtain stable SiNPR1 knockout or overexpression lines in order to fully elucidate the role of SiNPR1 in sesame against *R*. *solanacearum*.

## 4. Materials and Methods

### 4.1. Plant Materials

The field experiment was conducted in Jinxian County, Jiangxi Province, China (28° 23′ N; 116° 12′ E), using the sesame varieties Jinhuangma, Poyang Heizhima No. 5, Ganzhi No. 5, and Yuzhi No. 11, which were obtained from the Crop Research Institute of the Jiangxi Academy of Agricultural Sciences. The traditional sesame planting area is a field where sesame susceptible varieties have been continuously planted for 3 years, and the disease rate in the field reached 80% in the previous year. In order to apply exogenous hormones, 50 μg/mL BTH was sprayed on the leaves of sesame plants during the early flowering stage, and a control group was used that was sprayed with sterilized ddH_2_O. Bacterial wilt grading was performed according to the standards of Li et al. (2018) [33]. The experiment was not affected by extreme weather conditions.

*Ralstonia solanacearum* SEPPX05 were grown at 30 °C in BG medium (bacto peptone, 10 g/L; yeast extract, 1 g/L; casamino acids, 1 g/L; glucose, 5 g/L; pH 7.0). The bacterial indoor virulence experiment using drenching infection assays was adapted from Li et al. [33]. Seeds of the Poyang Heizhima No. 5 were sown in a commercial soil mix consisting of peat moss and perlite at a ratio of 2:1 by volume. The seeds were planted in plastic pots and maintained in a greenhouse at 25 °C, 60–70 mmol photons m^−2^ s^−1^, and a relative humidity of 70%, under a 16/8 h photoperiod. For the application of exogenous hormones, leaves of sesame plants at the four-leaf stage were sprayed with 50 µg/mL BTH, and a control group was used that was sprayed with sterilized ddH_2_O. Leaf samples were harvested from control and hormone-treated plants after 0, 12, 24 and 36 h for organ-specific expression analysis. All tissue samples were immediately frozen in liquid nitrogen and kept at −80 °C until further processing. Each biological sample was collected from three individual plants in this study.

*Nicotiana benthamiana* was grown in an artificial climate chamber under conditions including a 16 h light and 8 h dark cycle, at a temperature of 22–25 °C, with 4500 lux of supplemental light and a relative humidity of 50%.

### 4.2. Methods

#### 4.2.1. RNA Extraction and qRT-PCR Analysis

Total RNA was extracted from Sesame using an RNA-easy Isolation Reagent Kit (Vazyme, Nanjing, China), the purified total RNA (0.5–2 µg) was reverse transcribed into first-strand cDNA for RT-PCR and qRT-PCR analyses using the Reverse Transcription Kit (TaKaRa Bio, Beijing, China). The qRT-PCR analysis was performed using Hieff UNICON^®^ ColorGPS qPCR SYBR Green Master Mix (Yeasen Bio, Shanghai, China), and all experiments were repeated more than three times. Gene-specific primers were designed using Primer 7.0 (Table S5).

#### 4.2.2. Cloning of *SiNPR1* Gene Sequence

To clone the *SiNPR1* gene, complementary DNA (cDNA) from sesame leaf samples was prepared as a template. Specific primers were designed using Primer 7.0 (Table S5). The PCR products were purified by Agarose Gel Extraction Kit (TaKaRa Bio, Beijing, China), and the purified target gene products were ligated with the cloning vector pMD-19T (TaKaRa Bio, Beijing, China) and transformed into *Escherichia coli* DH5α (Sangon Biotech, Shanghai, China) in order to select a single colony for bacteriological PCR and sequencing (Sangon Biotech, Shanghai, China). The sequence comparison of target genes was performed by DNAMAN 8.0 software.

#### 4.2.3. Cloning of *SiNPR1* Promoter

The 5′-flanking region of *SiNPR1* was amplified using the BD Universal Genome Walker^TM^ kit (Clontech, Mountain View, CA, USA). The genomic DNA from sesame was digested with restriction enzymes, Dra I, EcoR V, Stu I, and Pvu II (TaKaRa Bio, Beijing, China) to generate blunt-end fragments, and subsequently the GenomeWalker Adaptors were ligated to the DNA fragments to generate four DNA libraries. Nested PCR was performed using the prepared DNA template along with the adaptor primers (AP1, AP2 provided by the kit) and gene specific primers (Table S5). The final purified PCR products were cloned into cloning vector pMD-19T (TaKaRa Bio, Beijing, China) and transformed into *Escherichia coli* DH5α (Sangon Biotech, Shanghai, China) in order to select a single colony for bacteriological PCR and sequencing (Sangon Biotech, Shanghai, China).

#### 4.2.4. Sequence Analysis and Gene Cloning of *SiTGA2*

To clone the full-length CDs fragment of *SiTGA2*, we designed a pair of primers (Table S5) based on the conserved regions of TGA2 orthologs and sesame (cultivar Zhongzhi No. 13) *TGA2* like sequence (XM_011080767.2), and complementary DNA (cDNA) from sesame leaf samples was prepared as a template. The PCR product was ligated with the cloning vector (TaKaRa Bio, Beijing, China) and transformed into *Escherichia coli* DH5α (Sangon Biotech, Shanghai, China) in order to select a single colony for bacteriological PCR and sequencing (Sangon Biotech, Shanghai, China). MEGA11 software (Version 11.0.13, Mega Limited, Auckland, New Zealand) was used to compare TGA2 homologous sequences and construct phylogenetic trees.

#### 4.2.5. Bioinformatics Analysis

The NCBI-ORF Finder was used to determine the ORF of the *SiNPR1* gene; the NCBI and SMART online programs were used to analyze the protein domain encoded by the *SiNPR1* gene (accessed on 18 October 2025). The physicochemical properties and hydrophobicity of the SiNPR1 protein were predicted using the Expasy ProtParam and Expasy ProtScale online programs, respectively (accessed on 18 October 2025). The potential phosphorylation site prediction analysis of the SiNPR1 protein was predicted using the NetPhosv 3.1 online program (accessed on 18 October 2025). The secondary structure and tertiary structure of the SiNPR1 protein were predicted using the SOPMA and SWISS-MODEL online programs (accessed on 18 October 2025). The signal peptides and transmembrane structures of the SiNPR1 protein were predicted using the SignalP 4.1 and TMHMM Server v.2.0 online programs, respectively (accessed on 18 October 2025). A BLAST search of homologous sequences of SiNPR1 proteins was conducted in the NCBI database (accessed on 18 October 2025), and MEGA11 software (Version 11.0.13, Mega Limited, Auckland, New Zealand) was used to compare homologous sequences and construct phylogenetic trees. The CELLO online program was used to predict the subcellular localization of the *SiNPR1* gene (accessed on 18 October 2025). The cis-element motifs of the gene were predicted using the PlantCARE online program (accessed on 18 October 2025). The specific websites are listed in Table S6 as Supplementary Data.

#### 4.2.6. Subcellular Localization

The *SiNPR1* gene was amplified using specific primers containing restriction sites (Table S5), and the obtained target genes were ligated into pCAMBIA3302 expression vectors containing the 35S promoter using T4 ligase via *Nco* I and *Spe* I enzyme digestion (TaKaRa Bio, Beijing, China). The empty vector served as a control. After sequence confirmation, these recombinant plasmids were transformed into *Agrobacterium tumefaciens* cells and were transiently expressed in the leaves of *N*. *benthamiana* plants at 5 weeks old as previously described [46]. At 48 h post-transformation, the infiltrated leaves were collected for the detection of fluorescent signals using a laser confocal microscope (SP8, Leica, Wetzlar, Germany). The emission and excitation wavelengths of GFP were set at 510–520 nm and 488 nm, respectively.

#### 4.2.7. Yeast Two-Hybrid (Y2H) Assays

Interactions between SiNPR1 and SiTGA2 were assessed using Y2H assays using the BD Matchmaker system (Clontech, Mountain View, CA, USA) as previously described [19]. Specifically, the complete open reading frame (ORF) and selected segments of SiNPR1 were cloned into the GAL4 DNA-binding domain pGBKT7 vector using *Nde* I (Takara, Dalian, China) and *BamH* I (Takara, Dalian, China). The complete ORF of SiTGA2 was inserted into the pGADT7 vector. These constructs were transformed into the AH109 yeast strain. Finally, co-transformants were plated on selective SD/TLHA (SD/-Trp/-Leu/-His/-Ade) medium with 20 mg/mL X-α-Gal (Sigma, St. Louis, MO, USA).

#### 4.2.8. Statistical Analysis

Statistical comparisons between two experimental groups were performed using Student’s *t*-test in GraphPad Prism 8.0. Error bars in the figures represent standard deviations (SD) as specified in the figure legends. All data are presented as the mean ± SD from at least three independent experiments. Results were considered statistically significant at *p* < 0.05. The specific significance levels (*: *p* < 0.05, **: 0.001 < *p* < 0.01, ***: 0.0001 < *p* < 0.001) are indicated directly in the figures with asterisks. All experiments were repeated three times.

## 5. Conclusions

In this study, the function, expression pattern, structural characteristics, and physicochemical properties of the sesame *NPR1* gene *SiNPR1* were analyzed. Phylogenetic analysis showed that the SiNPR1 amino acid sequence was clustered together with the reported NPR1s. Localization results suggest that SiNPR1 is located in the nucleus. *SiNPR1* was most highly expressed in sesame leaves, followed by the roots and stems. Expression of *SiNPR1* can be induced by BTH, and the pretreatment with BTH significantly increased the resistance of sesame to *R*. *solanacearum* and reduced the incidence. The yeast two-hybrid assay results indicated that SiNPR1 interacted with SiTGA2; however, whether to activate the expression of a number of *PR* genes and ultimately enhance the resistance of sesame to *R*. *solanacearum* needs to be confirmed. Therefore, this study provides a theoretical basis for the potential application of NPR1 in the improvement of sesame resistance to *R*. *solanacearum*, and is of great significance for sesame disease resistance breeding.

## Data Availability

All analyzed data from this study are included in the content of this paper and in the Supplementary Materials.

## Supplementary Materials

The following supporting information can be downloaded at: https://www.mdpi.com/article/10.3390/plants14233557/s1, Figure S1: Cloned cDNA fragment of *SiNPR1* gene; Figure S2: Autoactivation and empty vector tests; Table S1: Prediction of SiNPR1; Table S2: NPR1-like protein sequences from other plant species for phylogram construction; Table S3: *Cis*-elements identified in the promoter region of SiNPR1; Table S4: TGA2-like protein sequences from other plant species for phylogram construction; Table S5: The primers used in this study; Table S6: Web sites for bioinformatics analysis.

## References

[1] Pieterse C.M.J., Leon-Reyes A., Sjoerd V.D.E., Van Wees S.C.M. (2009). Networking by small-molecule hormones in plant immunity. Nat. Chem. Biol..

[2] An C., Mou Z. (2011). Salicylic acid and its function in plant immunity. J. Integr. Plant Biol..

[3] Fu Z.Q., Dong X. (2013). Systemic acquired resistance: Turning local infection into global defense. Annu. Rev. Plant Biol..

[4] David L., Harmon A.C., Chen S. (2019). Plant immune responses-from guard cells and local responses to systemic defense against bacterial pathogens. Plant Signal. Behav..

[5] Kim T.J., Lim G.H. (2023). Salicylic acid and mobile regulators of systemic immunity in plants: Transport and metabolism. Plants.

[6] Shine M., Xiao X., Kachroo P., Kachroo A. (2019). Signaling mechanisms underlying systemic acquired resistance to microbial pathogens. Plant Sci..

[7] Durrant W.E., Dong X. (2004). Systemic acquired resistance. Annu. Rev. Phytopathol..

[8] Yoshioka K., Nakashita H., Klessig D.F., Yamaguchi I. (2001). Probenazole induces systemic acquired resistance in *Arabidopsis* with a novel type of action. Plant J..

[9] Durner J., Klessig D.F. (1995). Inhibition of ascorbate peroxidase by salicylic acid and 2,6-dichloroisonicotinic acid, two inducers of plant defense responses. Proc. Natl. Acad. Sci. USA.

[10] Yalpani N., Raskin I. (1993). Salicylic acid: A systemic signal in induced plant disease resistance. Trends Microbiol..

[11] Cao H., Glazebrook J., Clarke J.D., Volko S., Dong X. (1997). The *Arabidopsis* NPR1 gene that controls systemic acquired resistance encodes a novel protein containing ankyrin repeats. Cell.

[12] Wang W., Withers J., Li H., Zwack P.J., Rusnac D.V., Shi H., Liu L., Yan S., Hinds T.R., Guttman M. (2020). Structural basis of salicylic acid perception by *Arabidopsis* NPR proteins. Nature.

[13] Seo S., Kim Y., Park K. (2023). NPR1 Translocation from Chloroplast to Nucleus Activates Plant Tolerance to Salt Stress. Antioxidants.

[14] Zavaliev R., Dong X. (2024). NPR1, a key immune regulator for plant survival under biotic and abiotic stresses. Mol. Cell.

[15] Li M., Li M., Qi S., Wang L., Kim C. (2025). Salicylic acid and ROS signaling modulate hypocotyl elongation in darkness via NPR1 and EX1. Sci. Adv..

[16] Eulgem T., Somssich I.E. (2007). Networks of WRKY transcription factors in defense signaling. Curr. Opin. Plant Biol..

[17] Rochon A., Boyle P., Wignes T., Fobert P.R., Despres C. (2006). The coactivator function of *Arabidopsis* NPR1 requires the core of its BTB/POZ domain and the oxidation of C-terminal cysteines. Plant Cell.

[18] Fan W., Dong X. (2002). In vivo interaction between NPR1 and transcription factor TGA2 leads to salicylic acid-mediated gene activation in *Arabidopsis*. Plant Cell.

[19] Sun L.M., Fang J.B., Zhang M., Qi X.J., Lin M.M., Chen J.Y. (2020). Molecular cloning and functional analysis of the NPR1 homolog in kiwifruit (*Actinidia eriantha*). Front. Plant Sci..

[20] Chern M., Fitzgerald H.A., Canlas P.E., Navarre D.A., Ronald P.C. (2005). Overexpression of a rice *NPR1* homolog leads to constitutive activation of defense response and hypersensitivity to light. Mol. Plant Microbe Interact..

[21] Liu X., Liu Z., Niu X., Xu Q., Yang L. (2019). Genome-wide identification and analysis of the *NPR*1-like gene family in bread wheat and its relatives. Int. J. Mol. Sci..

[22] Maldonado A., Youssef R., McDonald M., Brewer E., Beard H., Matthews B. (2014). Modification of the expression of two NPR1 suppressors, *SNC1* and *SNI1*, in soybean confers partial resistance to the soybean cyst nematode, *Heterodera glycines*. Funct. Plant Biol..

[23] Zhang J.Y., Qiao Y.S., Lv D., Gao Z.H., Qu S.C., Zhang Z. (2012). *Malus hupehensis* NPR1 induces pathogenesis-related protein gene expression in transgenic tobacco. Plant Biol..

[24] Yuan Y., Zhong S., Li Q., Zhu Z., Lou Y., Wang L., Wang J., Wang M., Li Q., Yang D. (2007). Functional analysis of rice *NPR1*-like genes reveals that OsNPR1/NH1 is the rice orthologue conferring disease resistance with enhanced herbivore susceptibility. Plant Biotechnol. J..

[25] Peng A., Zou X., He Y., Chen S., Liu X., Zhang J., Zhang Q., Xie Z., Long J., Zhao X. (2021). Overexpressing a *NPR1*-like gene from *Citrus paradisi* enhanced Huanglongbing resistance in *C. sinensis*. Plant Cell Rep..

[26] Zhang J., Jiao P., Zhang C., Tong X., Wei Q., Xu L. (2016). Apple *NPR1* homologs and their alternative splicing forms may contribute to SA and disease responses. Tree Genet. Genomes.

[27] Xing L., Gao L., Chen Q., Pei H., Wang X. (2018). Over-expressing a UDP-glucosyltransferase gene (*Ta-UGT_3_*) enhances *Fusarium* Head Blight resistance of wheat. Plant Growth Regul..

[28] Tada Y., Spoel S.H., Pajerowska-Mukhtar K., Mou Z., Song J., Wang C., Zuo J., Dong X. (2008). Plant immunity requires conformational charges of NPR1 via S-nitrosylation and thioredoxins. Science.

[29] Angamuthu M., Govindasamy S., Kasirajan S., Langyan S., Rangan P., Pradheep K. (2025). Origin and history of sesame and its uses. Sesame: Sustainable Production and Applications.

[30] Rauf S., Basharat T., Gebeyehu A., Elsafy M., Rahmatov M., Ortiz R., Kaya Y. (2024). Sesame, an underutilized oil seed crop: Breeding achievements and future challenges. Plants.

[31] Zhang H., Langham D.R., Miao H. (2021). Economic and academic importance of sesame. The Sesame Genome.

[32] Wang R., Li X., Lv F., He J., Lv R., Wei L. (2024). Sesame bacterial wilt significantly alters rhizosphere soil bacterial community structure, function, and metabolites in continuous cropping systems. Microbiol. Res..

[33] Li L., Rao J., Xiao Y., Wei L., Wang R., Huang R., Huang R., Hu J., Hua J. (2018). Identification of resistance of sesame (*Sesamum indicum*) germplasm resources to bacterial wilt (*Ralstorinia solanacearum*). Acta Agric. Jiangxi.

[34] Li X., Huang X., Chen G., Zou L., Wei L., Hua J. (2018). Complete genome sequence of the sesame pathogen *Ralstonia solanacearum* strain SEPPX 05. Genes Genom..

[35] Friedrich L., Lawton K., Ruess W., Masner P., Specker N., Rella M.G., Meier B., Dincher S., Staub T., Uknes S. (1996). A benzothiadiazole derivative induces systemic acquired resistance in tobacco. Plant J..

[36] Bovie C., Ongena M., Thonart P., Dommes J. (2004). Cloning and expression analysis of cDNAs corresponding to genes activated in cucumber showing systemic acquired resistance after BTH treatment. BMC Plant Biol..

[37] Deng H., Ma L., Gong D., Xue S., Ackah S., Prusky D., Bi Y. (2023). BTH-induced joint regulation of wound healing at the wounds of apple fruit by JA and its downstream transcription factors. Food Chem..

[38] Li H., Wu J., Shang X., Geng M., Gao J., Zhao S., Yu X., Liu D., Kang Z., Wang X. (2020). *WRKY* transcription factors shared by BTH-induced resistance and *NPR1*-mediated acquired resistance improve broad-spectrum disease resistance in wheat. Mol. Plant Microbe Interact..

[39] Ding Y., Sun T., Ao K., Peng Y., Zhang Y., Li X., Zhang Y. (2018). Opposite roles of salicylic acid receptors NPR1 and NPR3/NPR4 in transcriptional regulation of plant immunity. Cell.

[40] Zhang Y., Fan W., Kinkema M., Li X., Dong X. (1999). Interaction of NPR1 with basic leucine zipper protein transcription factors that bind sequences required for salicylic acid induction of the *PR-1* gene. Proc. Natl. Acad. Sci. USA.

[41] Boyle P., Le Su E., Rochon A., Shearer H.L., Murmu J., Chu J.Y., Fobert P.R., Despres C. (2009). The BTB/POZ domain of the *Arabidopsis* disease resistance protein NPR1 interacts with the repression domain of TGA2 to negate its function. Plant Cell.

[42] Johnson C., Mhatre A., Arias J. (2008). NPR1 preferentially binds to the DNA-inactive form of *Arabidopsis* TGA2. Biochim. Biophys. Acta.

[43] Mou Z., Fan W., Dong X. (2003). Inducers of plant systemic acquired resistance regulate *NPR1* function through redox changes. Cell.

[44] Le Henanff G., Heitz T., Mestre P., Mutterer J., Walter B., Chong J. (2009). Characterization of *Vitis vinifera* NPR1 homologs involved in the regulation of pathogenesis-related gene expression. BMC Plant Biol..

[45] Zang S., Qin L., Zhao Z., Zhang J., Zou W., Wang D., Feng A., Yang S., Que Y., Su Y. (2022). Characterization and functional implications of the Nonexpressor of Pathogenesis-Related genes 1 (*NPR1*) in *Saccharum*. Int. J. Mol. Sci..

[46] Zhong X., Xi L., Lian Q., Luo X., Wu Z., Seng S., Yuan X., Yi M. (2015). The *NPR1* homolog *GhNPR1* plays an important role in the defense response of *Gladiolus hybridus*. Plant Cell Rep..

[47] Frackowiak P., Pospieszny H., Smiglak M., Obrepalska-Steplowska A. (2019). Assessment of the efficacy and mode of action of benzo(1,2,3)-thiadiazole-7-carbothioic acid s-methyl ester (BTH) and its derivatives in plant protection against viral disease. Int. J. Mol. Sci..

[48] Achuo A.E., Hofte M. (2001). Potential of induced resistance to control *Oidium lycopersici* on tomato and tobacco. Mededelingen.

[49] Cheng Z., Yu X., Li S., Wu Q. (2018). Genome-wide transcriptome analysis and identification of benzothiadiazole-induced genes and pathways potentially associated with defense response in banana. BMC Genom..

