# Supplementary material for: Molecular Cloning, Bioinformatics, and Expression Analysis of the NPR1 Homolog in Sesame (Sesamum indicum L.)
Source: Plants (Basel). 2025 Nov 21;14(23):3557. doi: 10.3390/plants14233557 (PMC12693970; doi:10.3390/plants14233557)
Supplement: Supplementary file 1 [file plants-14-03557-s001.zip › Supplementary Figure S1. Cloned cDNA fragment of SiNPR1 gene.pdf]

Supplementary Figure S1 Cloned cDNA fragment of *SiNPR1* gene

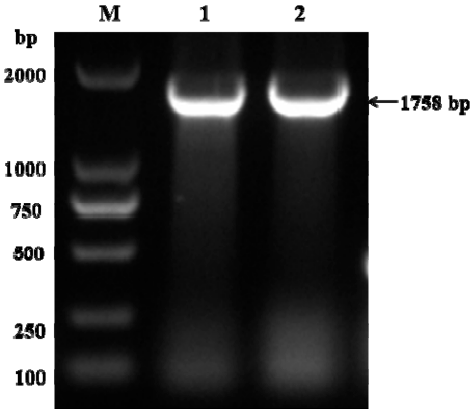

Figure S1. Cloned cDNA fragment of *SiNPR1* gene. M: Mark DL 2000, 1, 2: SiNPR1 gene.
