# Supplementary material for: Molecular Cloning, Bioinformatics, and Expression Analysis of the NPR1 Homolog in Sesame (Sesamum indicum L.)
Source: Plants (Basel). 2025 Nov 21;14(23):3557. doi: 10.3390/plants14233557 (PMC12693970; doi:10.3390/plants14233557)
Supplement: Supplementary file 1 [file plants-14-03557-s001.zip › Supplementary Figure S2. Autoactivation and empty vector tests.pdf]

Supplementary Figure S2 Autoactivation and empty vector tests

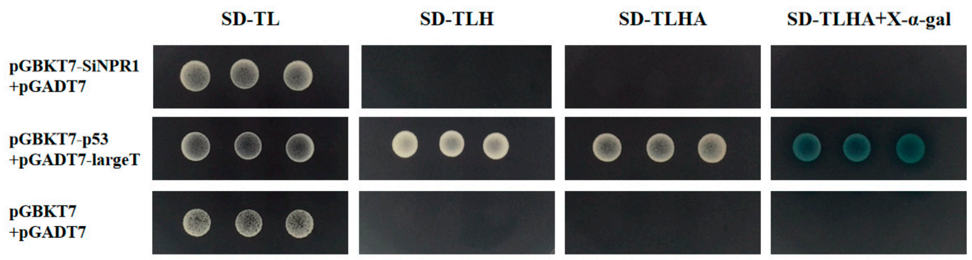

Figure S2. Autoactivation and empty vector tests. Transformed yeast cells were diluted with 0.9% NaCl into three concentration gradients (10^{-1}, 10^{-2}, and 10^{-3}), and streaked on SD/TL (SD/-Trp/-Leu), SD/TLHA (SD/-Trp/-Leu/-His), SD/TLHA (SD/-Trp/-Leu/-His/-Ade), and SD/TLHA (SD/-Trp/-Leu/-His/-Ade) medium containing 20 mg/ml X-α-Gal.
