# Supplementary material for: Molecular Cloning, Bioinformatics, and Expression Analysis of the NPR1 Homolog in Sesame (Sesamum indicum L.)
Source: Plants (Basel). 2025 Nov 21;14(23):3557. doi: 10.3390/plants14233557 (PMC12693970; doi:10.3390/plants14233557)
Supplement: Supplementary file 1 [file plants-14-03557-s001.zip › Supplementary Table S1. Prediction of SiNPR1.pdf]

Supplementary Table S1. Prediction of SiNPR1.

Support vector machine	Localization	Reliability
Amino Acid comp.	Nuclear	0.501
N-peptide Comp.	Cytoplasmic	0.325
Partitioned seg. Comp.	Nuclear	0.789
Physico-chemical Comp.	Cytoplasmic	0.575
Neighboring seg. Comp.	Cytoplasmic	0.405
