# Supplementary material for: Molecular Cloning, Bioinformatics, and Expression Analysis of the NPR1 Homolog in Sesame (Sesamum indicum L.)
Source: Plants (Basel). 2025 Nov 21;14(23):3557. doi: 10.3390/plants14233557 (PMC12693970; doi:10.3390/plants14233557)
Supplement: Supplementary file 1 [file plants-14-03557-s001.zip › Supplementary Table S2. NPR1-like protein sequences from other plant species for phylogram construction.pdf]

Supplementary Table S2. NPR1-like protein sequences from other plant species for phylogram construction.

Identifier	Accession number	Species	Lineage
OeNPR1	CAA3012024.1	*Olea europaea*	Dicotyledon
CcNPR1	CAP12787.1	*Capsicum chinense*	Dicotyledon
NtNPR1	AAM62410.1	*Nicotiana tabacum*	Dicotyledon
GhNPR1	NP_001386783.1	*Gossypium hirsutum*	Dicotyledon
AtNPR1	NP_176610.1	*Arabidopsis thaliana*	Dicotyledon
LeNPR1	APY24056.1	*Solanum lycopersicum*	Dicotyledon
GmNPR1-1	ACJ45013.1	*Glycine max*	Dicotyledon
GmNPR1-2	ACJ45015.1	*Glycine max*	Dicotyledon
AtNPR2	NP_001329623.1	*A. thaliana*	Dicotyledon
AtNPR3	NP_001330386.1	*A. thaliana*	Dicotyledon
AtNPR4	NP_001328027.1	*A. thaliana*	Dicotyledon
AcNPR1	PSS20797.1	*Actinidia chinensis*	Dicotyledon
IbNPR1	ABM64782.1	*Ipomoea batatas*	Dicotyledon

CaNPR1	ABG38308.1	*Capsicum annuum*	Dicotyledon
StNPR1	XP_006357709.1	*Solanum tuberosum*	Dicotyledon
CpNPR1	AAS55117.1	*Carica papaya*	Dicotyledon
VvNPR1.1	XP_002281475.1	*Vitis vinifera*	Dicotyledon
VvNPR1.2	XP_003633057.1	*Vitis vinifera*	Dicotyledon
CsNPR1	XP_010418483.1	*Camelina sativa*	Dicotyledon
CsNPR3	XP_010494583.1	*C. sativa*	Dicotyledon
EgNPR1	XP_010915286.2	*Elaeis guineensis*	Monocotyledon
EgNPR3	XP_010908601.1	*E. guineensis*	Monocotyledon
PdNPR1	XP_008782513.1	*Phoenix dactylifera*	Monocotyledon
PdNPR3	XP_008806697.1	*P. dactylifera*	Monocotyledon
GlNPR1	AIM54370.1	*Gladiolus hybrid*	Monocotyledon
ZmNPR1	DAA52994.1	*Zea mays*	Monocotyledon
OsNPR1	AAX18700.1	*Oryza sativa*	Monocotyledon
TdNPR1	AGH18701.1	*Triticum durum*	Monocotyledon
SinNPR1.1	XP_011078003.1	*Sesamum indicum*	Dicotyledon

SaNPR1	KAK4432864.1	*S. alatum*	Dicotyledon
SinNPR1.2	XP_011078001.1	*S. indicum*	Dicotyledon
SiNPR1	PX427686.1	*S. indicum*	Dicotyledon
