# Supplementary material for: Molecular Cloning, Bioinformatics, and Expression Analysis of the NPR1 Homolog in Sesame (Sesamum indicum L.)
Source: Plants (Basel). 2025 Nov 21;14(23):3557. doi: 10.3390/plants14233557 (PMC12693970; doi:10.3390/plants14233557)
Supplement: Supplementary file 1 [file plants-14-03557-s001.zip › Supplementary Table S3. Cis-elements identified in the promoter region of SiNPR1.pdf]

Supplementary Table S3. *Cis*-elements identified in the promoter region of SiNPR1.

Regulated element	Motif sequence	Amount	Biological function
TGA-element	AACGAC	1	auxin-responsive element
ABRE	ACGTG	2	abscisic acid responsiveness
CGTCA-motif	CGTCA	1	methyl jasmonate responsiveness
G-Box	CACGTT	2	light responsiveness
LTR	CCGAAA	1	low-temperature responsiveness
TATC-box	TATCCCA	1	gibberellin responsiveness
Box 4	ATTAAT	3	light responsiveness
TATA-box	TATA/TATAA/TA TAAATA/ATATAT	102	core promoter element around -30 of transcription start
CAAT-box	CAAAT/CCCAAT TT/CCAAT	16	common cis-acting element in promoter and enhancer regions
TGACG-motif	TGACG	1	methyl jasmonate responsiveness
AT-rich sequence	TAAAATACT	1	element for maximal elicitor-mediated activation
I-box	AGATAAGG	1	light responsive element
WUN-motif	AAATTTCCT	1	wound responsive element
