# Supplementary material for: Molecular Cloning, Bioinformatics, and Expression Analysis of the NPR1 Homolog in Sesame (Sesamum indicum L.)
Source: Plants (Basel). 2025 Nov 21;14(23):3557. doi: 10.3390/plants14233557 (PMC12693970; doi:10.3390/plants14233557)
Supplement: Supplementary file 1 [file plants-14-03557-s001.zip › Supplementary Table S4. TGA2-like protein sequences from other plant species for phylogram construction.pdf]

Supplementary Table S4. TGA2-like protein sequences from other plant species for phylogram construction.

Identifier	Accession number	Species	Lineage
OeTGA2	CAA3029065.1	*Olea europaea*	Dicotyledon
GhTGA2	KAG4183224.1	*Gossypium hirsutum*	Dicotyledon
AtTGA2	NP_001078539.1	*Arabidopsis thaliana*	Dicotyledon
PvTGA2	XP_031285443.1	*Pistacia vera*	Dicotyledon
VvTGA2	XP_002263159	*Vitis vinifera*	Dicotyledon
NtTGA2	NP_001312154.1	*Nicotiana tabacum*	Dicotyledon
TcTGA2	XP_017977404.1	*Theobroma cacao*	Dicotyledon
PtTGA2	XP_002303938.4	*Populus trichocarpa*	Dicotyledon
AtTGA1	NP_001318881.1	*Arabidopsis thaliana*	Dicotyledon
AtTGA3	NP_564156.1	*Arabidopsis thaliana*	Dicotyledon
AtTGA4	NP_001330616.1	*Arabidopsis thaliana*	Dicotyledon
CaTGA2	KAF3612957.1	*Capsicum annuum*	Dicotyledon
SaTGA2	KAK4392527.1	*Sesamum angolense*	Dicotyledon
AcTGA2	PSS33519.1	*Actinidia chinensis*	Dicotyledon
OsTGA2	BAS83919.1	*Oryza sativa*	Monocotyledon
