# Supplementary material for: Molecular Cloning, Bioinformatics, and Expression Analysis of the NPR1 Homolog in Sesame (Sesamum indicum L.)
Source: Plants (Basel). 2025 Nov 21;14(23):3557. doi: 10.3390/plants14233557 (PMC12693970; doi:10.3390/plants14233557)
Supplement: Supplementary file 1 [file plants-14-03557-s001.zip › Supplementary Table S5. The primers used in this study.pdf]

**Supplementary Table S5. The primers used in this study.**

| Primer name | Primer sequence (5′ to 3′) |
|---|---|
| The RACE primers for the *SiNPR1* gene amplication | |
| NPR1-1-F | CCCCTTATAGTAGAGTGC |
| NPR1-1-R | CATCACCTCCTTCAGC |
| NPR1-2-F | GCTGAAGGAGGTGATGAA |
| NPR1-2-R | TAGCAGGAAATGGGTGTT |
| NPR1-3-F | CTTTCACCGAACACCCAT |
| NPR1-3-R | CACGCAACTCTTCCATCC |
| NPR1-4-F | GACTTTGGGATATTACTCATT |
| NPR1-4-R | AATGGATTTATTGGTCTCTA |
| The primers for the *SiNPR1* gene ORF amplication | |
| SiNPR1-F | ATGGGTAGTCGAACCGCATT |
| SiNPR1-R | TCAGTTCGTGAAGGTGAGATTGC |

Expression of *SiNPR1* based pCAMBIA3302

| | |
|---|---|
| SiNPR1-F *Nco* I | TCTTGACCATGGGTAGTCGAACCGCATT |
| SiNPR1-R *Spe* I | CCTTTACTAGTCAGTTCGTGAAGGTGAGATTGC |

The primers for the *SiTGA2* gene ORF amplication

| | |
|---|---|
| SiTGA2-F | ATGGGTAGTAGAACAGCTAAAATTG |
| SiTGA2-R | TCATTCTCGTGGCCTTGCAAGCC |

The primers for the *SiNPR1* promoter amplication

| | |
|---|---|
| AP1 | GTAATACGACTCACTATAGGGC |
| AP2 | ACTATAGGGCACGCGTGGT |
| SiNPR1-GSP1 | TCGATGTTAAAATATCAATTCTCATTCA |
| SiNPR1-GSP2 | TGCCATTTAATTATAGCAGGCACTCTACT |

The primers for yeast two-hybrid assay

| | |
|---|---|
| SiNPR1-BD1-F | TCTGACATATGGGTAGTCGAACCGCATT |
| SiNPR1-BD1-R | CTTGTGGATCCTCAGTTCGTGAAGGTGAGATTGC |

| | |
|---|---|
| SiNPR1-BD2-R | ACTTGT<u>GGATCC</u>TCAATCAGATGGTCTAGCTCCCTT |
| SiNPR1-BD3-R | CTTGT<u>GGATCC</u>TCAGTCAAGAATCATCATCAG |
| SiNPR1-BD4-F | TCTGA<u>CATATG</u>CTCACTTCAGATGGCCGAAAAG |
| SiNPR1-BD4-R | CTTGT<u>GGATCC</u>TCAGTTCGTGAAGGTGAGATTG |
| SiTGA2-AD-F | TCCA<u>CCCGGG</u>TATGGGTAGTAGAACAGCTAAAATTG |
| SiTGA2-AD-R | GCTCGAT<u>GGATCC</u>TCATTCTCGTGGCCTTGCAAGCC |
| The primers for Real-time quantitatively RT-PCR | |
| SiNPR1-qF | GAACTTCTTGACCTAGC |
| SiNPR1-qR | ATAAATGAGTCAAGGGTAGA |
| UBQ5-qF | TGGACACTCTTTCCTCAACCTCT |
| UBQ5-qR | TCTCGCCGACTACAACATTCA |
| Plasmid detection primer | |
| pMD-19T_detect F | GTTGTAAAACGACGGCCAG |

| | |
|---|---|
| pMD-19T_detect R | CAGGAAACAGCTATGAC |
| pCAMBIA3302_ F | GTTGTAAAACGACGGCCAG |
| pCAMBIA3302_ R | CAGGAAACAGCTATGAC |
| pGBKT7_detect F | TAATACGACTCACTATAGG |
| pGBKT7_detect R | CAGGAAACAGCTATGAC |
| pGADT7_detect F | TAATACGACTCACTATAGG |
| pGADT7_detect R | AGATGGTGCACGATGCACAG |

[a] Underlined nucleotide sequences are digestion sites of restriction endonucleases.
