# Supplementary material for: Molecular Cloning, Bioinformatics, and Expression Analysis of the NPR1 Homolog in Sesame (Sesamum indicum L.)
Source: Plants (Basel). 2025 Nov 21;14(23):3557. doi: 10.3390/plants14233557 (PMC12693970; doi:10.3390/plants14233557)
Supplement: Supplementary file 1 [file plants-14-03557-s001.zip › Supplementary Table S6. Web sites for bioinformatics analysis.pdf]

Supplementary Table S6. Web sites for bioinformatics analysis.

Tools	website
NCBI-ORF Finder	https://www.ncbi.nlm.nih.gov/orffinder/
SMART	https://smart.embl.de/
PlantCARE	http://bioinformatics.psb.ugent.be/webtools/plantcare/html/
Expasy ProtScale	https://web.expasy.org/protscale/
Expasy ProtParam	https://web.expasy.org/protparam/
NetPhos 3.1	https://services.healthtech.dtu.dk/services/NetPhos-3.1/
SOPMA	https://npsa-prabi.ibcp.fr/cgi-bin/npsa_automat.pl?page=npsa_sopma.html
SWISS-MODEL	http://swissmodel.expasy.org
SignalP 4.1	https://services.healthtech.dtu.dk/services/SignalP-4.1/
TMHMM Server v.2.0	http://www.cbs.dtu.dk/services/TMHMM/
NCBI database	https://www.ncbi.nlm.nih.gov/
CELLO	http://cello.life.nctu.edu.tw
